# Genome Sequence of *Salmonella enterica* Serovar Typhi Oral Vaccine Strain Ty21a

**DOI:** 10.1128/genomeA.00650-13

**Published:** 2013-08-22

**Authors:** DeQi Xu, John O. Cisar, Frédéric Poly, Jinghua Yang, Jason Albanese, Madushini Dharmasena, Tint Wai, Patricia Guerry, Dennis J. Kopecko

**Affiliations:** Laboratory of Enteric and Sexually Transmitted Diseases, Center for Biologics Evaluation and Research, U.S. Food and Drug Administration, Bethesda, Maryland, USAa; Microbial Receptors Section, Laboratory of Cell and Developmental Biology, National Institute of Dental and Craniofacial Research, National Institutes of Health, Bethesda, Maryland, USAb; Enteric Diseases Department, Naval Medical Research Center, Silver Spring, Maryland, USAc

## Abstract

Attenuated *Salmonella* *enterica* serovar Typhi strain Ty21a is an important vaccine for controlling typhoid fever and serves as an oral vector for delivering heterologous antigens. The key attenuating features of this randomly mutated strain remain in question. Genome sequencing has revealed 679 single nucleotide polymorphisms (SNPs), and will help define alterations contributing to Ty21a safety and immunogenicity.

## GENOME ANNOUNCEMENT

Prior genomic sequencing of *Salmonella enterica* serovar Typhi (1–3) has both stimulated research and increased our understanding of typhoid pathogenesis. Nevertheless, typhoid fever remains a major cause of worldwide morbidity and mortality, with an estimated global annual incidence of 22 million cases and >600,000 deaths (4, 5). The observed increasing antibiotic resistance of typhoid isolates in nature leaves typhoid vaccination as a critically important control measure.

Development of the live, attenuated *Salmonella enterica* serovar Typhi strain Ty21a (6) validated the concept that orally delivered, attenuated enteric bacteria can mimic natural infection in stimulating both mucosal and systemic immune responses without causing disease symptoms (7). Ty21a has been shown to be highly safe (i.e., no reversion to virulence) following administration to >200 million recipients over >25 years. Ty21a, tested in several large clinical trials totaling >500,000 adults and children, has demonstrated overall typhoid protective efficacies of 67 to 96%, which were maintained at high levels through at least 7 full years (8). Importantly, Ty21a attenuation resulted in an enviable balance of reduced virulence and enhanced immunogenicity relative to immunity derived following natural typhoid infections (i.e., only 30 to 35% protection following acute typhoid disease). Ty21a has also been employed as an oral delivery system to protect against other disease agents, such as shigellosis (9–11) and the biodefense threat anthrax (12).

Derived from parent strain Ty2, Ty21a was primarily mutated to *galE* negativity and Vi-capsule nonexpression by random mutagenic methods (6) but was also selected for downregulation of other genes in galactose metabolism (13, 14). Additionally, other mutations (e.g., in *rpoS* and *ilvD*) are also known to exist in Ty21a (14). A full accounting of Ty21a genomic mutations has, to date, been lacking. Thus, the intent of these studies has been to enumerate, locate, and analyze all mutations in Ty21a relative to the Ty2 parent. The resulting data are being used to gain a better understanding of Ty21a attenuation (i.e., safety) and enhanced immunogenicity over wild-type strains, information that might be helpful in the development of new attenuated bacterial vaccines.

DNA was prepared with a genomic isolation kit (Promega) from L-broth-grown Ty21a obtained from commercially available Vivotif capsules (Crucell/Berna Biotech, Ltd., Switzerland). Initially, a 98% complete Ty21a genomic sequence was derived using a NimbleGen chip-based (high-density microarray) resequencing analysis compared to the published Ty2 genome (NCBI accession number AE014613); this approach resulted in numerous uncalled nucleotides. Separately, a Ty21a genomic sequence obtained from 4× coverage by random 454-based sequencing was determined. These two sequences were used integratively to verify identical genomic regions and to detect areas of disparity. All incongruous regions were resequenced using double-strand sequence analysis for clarification. The Ty21a genome comprises a single circular contig consisting of 4,791,958 bp, with an average G+C content of 52.06%. A total of 4,339 coding regions (open reading frames [ORFs]) were defined in Ty21a using GeneMark (http://opal.biology.gatech.edu/GeneMark) and compared with known Ty2 ORFs by BLAST analysis. A total of 679 SNPs was identified in Ty21a, relative to the published Ty2 genome (2). A detailed analysis of these mutations will be included in future publications.

### Nucleotide sequence accession number. 

The *Salmonella ennterica* serovar Typhi strain Ty21a genome sequence has been deposited in GenBank under the accession number CP002099.

## References

[1] Parkhill J Dougan G James KD Thomson NR Pickard D Wain J Churcher C Mungall KL Bentley SD Holden MT Sebaihia M Baker S Basham D Brooks K Chillingworth T Connerton P Cronin A Davis P Davies RM Dowd L White N Farrar J Feltwell T Hamlin N Haque A Hien TT Holroyd S Jagels K Krogh A Larsen TS Leather S Moule S O’Gaora P Parry C Quail M Rutherford K Simmonds M Skelton J Stevens K Whitehead S Barrell BG 2001 Complete genome sequence of a multiple drug resistant *Salmonella enterica* serovar Typhi CT18. Nature 413:848–852. 10.1038/35101607 11677608

[2] Deng W Liou SR Plunkett G III Mayhew GF Rose DJ Burland V Kodoyianni V Schwartz DC Blattner FR 2003 Comparative genomics of *Salmonella enterica* serovar Typhi strains Ty2 and CT18. J. Bacteriol. 185:2330–2337 12644504 10.1128/JB.185.7.2330-2337.2003 PMC151493

[3] Holt KE Parkhill J Mazzoni CJ Roumagnac P Weill FX Goodhead I Rance R Baker S Maskell DJ Wain J Dolecek C Achtman M Dougan G 2008 High-throughput sequencing provides insights into genome variation and evolution in *Salmonella* Typhi. Nat. Genet. 40:987–993. 10.1038/ng.195 18660809 PMC2652037

[4] Crump JA Luby SP Mintz ED 2004 The global burden of typhoid fever. Bull. World Health Organ. 82:346–353 15298225 PMC2622843

[5] Crump JA Mintz ED 2010 Global trends in typhoid and paratyphoid fever. Clin. Infect. Dis. 50:241–246. 10.1086/649541 20014951 PMC2798017

[6] Germanier R Furer E 1975 Isolation and characterization of Gal E mutant Ty21a of *Salmonella typhi*: a candidate strain for a live typhoid vaccine. J. Infect. Dis. 141:553–558 1092768 10.1093/infdis/131.5.553

[7] McGhee JR Mestecky J Dertzbaugh MT Eldridge JH Hirasawa M Kiyono H 1992 The mucosal immune system: from fundamental concepts to vaccine development. Vaccine 10:75–88. 10.1016/0264-410X(92)90021-B 1539467

[8] Levine MM Plotkin SA Orenstein WA 1999 Vaccines, p 781–814 W. B. Saunders, Philadelphia, PA.

[9] Xu DQ Cisar JO Ambulos N Jr Burr DH Kopecko DJ 2002 Molecular cloning and characterization of genes for *Shigella sonnei* form I O polysaccharide: proposed biosynthetic pathway and stable expression in a live *Salmonella* vaccine vector. Infect. Immun. 70:4414–4423. 10.1128/IAI.70.8.4414-4423.2002 12117952 PMC128211

[10] Xu DQ Cisar JO Osorio M Wai TT Kopecko DJ 2007 Core-linked LPS expression of *Shigella dysenteriae* serotype 1 O-antigen in live *Salmonella* Typhi vaccine vector Ty21a: preclinical evidence of immunogenicity and protection. Vaccine 25:6167–6175. 10.1016/j.vaccine.2007.06.003 17629369

[11] Dharmasena MN Hanisch BW Wai TT Kopecko DJ 2013 Stable expression of *Shigella sonnei* form I O-polysaccharide genes recombineered into the chromosome of live *Salmonella* oral vaccine vector Ty21a. Int. J. Med. Microbiol. 303:105–113. 10.1016/j.ijmm.2013.01.001 23474241

[12] Osorio M Wu Y Singh S Merkel TJ Bhattacharyya S Blake MS Kopecko DJ 2009 Anthrax protective antigen delivered by *Salmonella enterica* serovar Typhi Ty21a protects mice from a lethal anthrax spore challenge. Infect. Immun. 77:1475–1482. 10.1128/IAI.00828-08 19179420 PMC2663156

[13] Cryz SJ Jr 1988 Attenuated, live, oral typhoid vaccine. Drugs Today 24:349–353

[14] Kopecko DJ Sieber H Ures JA Fürer A Schlup J Knof U Collioud A Xu D Coburn K Dietrich G 2009 Genetic stability of vaccine strain *Salmonella* Typhi Ty21a over 25 years. Int. J. Med. Microbiol. 299:233–246. 10.1016/j.ijmm.2008.09.003 19121604

